# Optically Induced Calcium-Dependent Gene Activation and Labeling of Active Neurons Using CaMPARI and Cal-Light

**DOI:** 10.3389/fnsyn.2019.00016

**Published:** 2019-05-24

**Authors:** Christian Ebner, Julia Ledderose, Timothy A. Zolnik, Sina E. Dominiak, Paul Turko, Athanasia Papoutsi, Panayiota Poirazi, Britta J. Eickholt, Imre Vida, Matthew E. Larkum, Robert N. S. Sachdev

**Affiliations:** ^1^NeuroCure Cluster of Excellence, Charité—Universitätsmedizin Berlin, Berlin, Germany; ^2^Institute for Biology, Humboldt-Universität zu Berlin, Berlin, Germany; ^3^Institute for Biochemistry, Charité—Universitätsmedizin Berlin, Berlin, Germany; ^4^Institute for Integrative Neuroanatomy, Charité—Universitätsmedizin Berlin, Berlin, Germany; ^5^Institute of Molecular Biology and Biotechnology, Foundation for Research and Technology—Hellas, Heraklion, Greece

**Keywords:** CaMPARI, Cal-Light, photoconversion, photoactivation, calcium, optogenetics, gene expression

## Abstract

The advent of optogenetic methods has made it possible to use endogeneously produced molecules to image and manipulate cellular, subcellular, and synaptic activity. It has also led to the development of photoactivatable calcium-dependent indicators that mark active synapses, neurons, and circuits. Furthermore, calcium-dependent photoactivation can be used to trigger gene expression in active neurons. Here we describe two sets of protocols, one using CaMPARI and a second one using Cal-Light. CaMPARI, a calcium-modulated photoactivatable ratiometric integrator, enables rapid network-wide, tunable, all-optical functional circuit mapping. Cal-Light, a photoactivatable calcium sensor, while slower to respond than CaMPARI, has the capacity to trigger the expression of genes, including effectors, activators, indicators, or other constructs. Here we describe the rationale and provide procedures for using these two calcium-dependent constructs (1) *in vitro* in dissociated primary neuronal cell cultures (CaMPARI & Cal-Light); (2) *in vitro* in acute brain slices for circuit mapping (CaMPARI); (3) *in vivo* for triggering photoconversion or gene expression (CaMPARI & Cal-Light); and finally, (4) for recovering photoconverted neurons post-fixation with immunocytochemistry (CaMPARI). The approaches and protocols we describe are examples of the potential uses of both CaMPARI & Cal-Light. The ability to mark and manipulate neurons that are active during specific epochs of behavior has a vast unexplored experimental potential.

## Introduction

A fundamental goal of neuroscience research is to understand what the activity of neurons represents: is the activity correlated with a particular sensory input or to a particular behavior? Do the neurons involved in learning, memory or behavior express specific markers or genetic programs that are activated during the learning or consolidation phase? To study why some neurons are active while neighboring neurons are inactive, or why some neurons show genetic changes during learning or consolidation or memory formation, requires a detailed understanding of their input and their genetic and physiological properties. To begin such analysis, we first need to identify a population of active neurons *in vivo* that can then be targeted with additional methods (DeNardo and Luo, 2017) to further interrogate their biophysical and genetic properties *in vitro*. To achieve this level of understanding, the ability to mark and track active neurons and recover them for additional experiments and analysis is essential.

Immediate early gene (IEG) expression has provided means to recover active neurons in experimental paradigms for decades. IEGs show low expression when cells are quiescent but stimulation can elicit transient high expression within minutes (Greenberg et al., 1986; Morgan and Curran, 1986; Kawashima et al., 2014; Yap and Greenberg, 2018). IEG expressing cells can be tracked online with fluorescence indicators or *post-hoc* by immunocytochemistry in fixed tissue. While IEG expression has proven useful, it has become clear that triggers for transcription of IEGs include metabolic activity, stress, growth factors, and the release of neurotransmitters (Sheng and Greenberg, 1990). Thus, the expression of those genes is not necessarily uniformly or tightly linked to activity, such as spiking or synaptic input. There have also been multiple forays into chemically induced gene expression (Mansuy et al., 1998; Dogbevia et al., 2015; Cazzulino et al., 2016). While these methods have been successfully applied to a variety of experimental paradigms, other recent approaches are both more versatile and temporally more precise. These new approaches can track active neurons *in vivo* and recover them for *ex vivo* experiments (Barth, 2007; Wang et al., 2019). They rely on optical measurements or photoconversion of calcium indicator dyes to tag activity of neurons with two-photon imaging, followed by *in vitro* recording from the same neurons (Ko et al., 2011), photoactivatable GFP for targeting neurons *in vivo* and *in vitro* (Lien and Scanziani, 2011; Peter et al., 2013) and chronic imaging with virally expressed GCaMP and fluorescent beads that make it possible to identify the set of neurons for *ex vivo* slice work (Weiler et al., 2018). These developments all point toward the need for developing simple methods to track active neurons over longer periods (over days) or from one experimental condition to another (e.g., from *in vivo* to *in vitro* experiments). Here, we describe procedures to make use of two calcium-dependent tools: CaMPARI and Cal-Light. Both can be used for marking active neurons, circuit mapping or optogenetic manipulations.

CaMPARI (calcium-modulated photoactivatable ratiometric integrator) is a calcium indicator that can be rapidly photoconverted in active neurons to perform circuit mapping (Fosque et al., 2015; Zolnik et al., 2017; Moeyaert et al., 2018). CaMPARI works well with channelrhodopsin (ChR2)-based circuit mapping because 405 nm light triggers both photoconversion of CaMPARI and activation of ChR2-positive neurons and axon terminals.

Cal-Light is another photoactivatable calcium-sensitive indicator that is able to trigger the expression of a variety of genes in active neurons (Lee et al., 2017). This property of Cal-Light allows for the selective expression of genes in active neurons and therefore can be used to interrogate whether the activity of these neurons is necessary and/or sufficient for a given behavior when driving the expression of constructs such as optogenetic silencers or enhancers.

Both CaMPARI and Cal-Light require illumination, coincident to the cytosolic calcium increase, to trigger conversion or activation, respectively. However, CaMPARI and Cal-Light operate on different time scales: CaMPARI converts rapidly within seconds (Fosque et al., 2015; Zolnik et al., 2017), whereas Cal-Light-dependent expression takes about 2–5 days (Lee et al., 2017). CaMPARI conversion and in principle Cal-Light activation can be used with ChR2 activation, since CaMPARI uses 405 nm light and Cal-Light 470 nm light, both of which can activate ChR2. Cal-Light offers the possibility to then trigger the expression of other constructs such as fluorescent proteins or optogenetic activators/inhibitors (e.g., GFP, ARChT, iChloC, etc.) in active neurons.

### Mechanisms of CaMPARI Action

**Figure 1:**
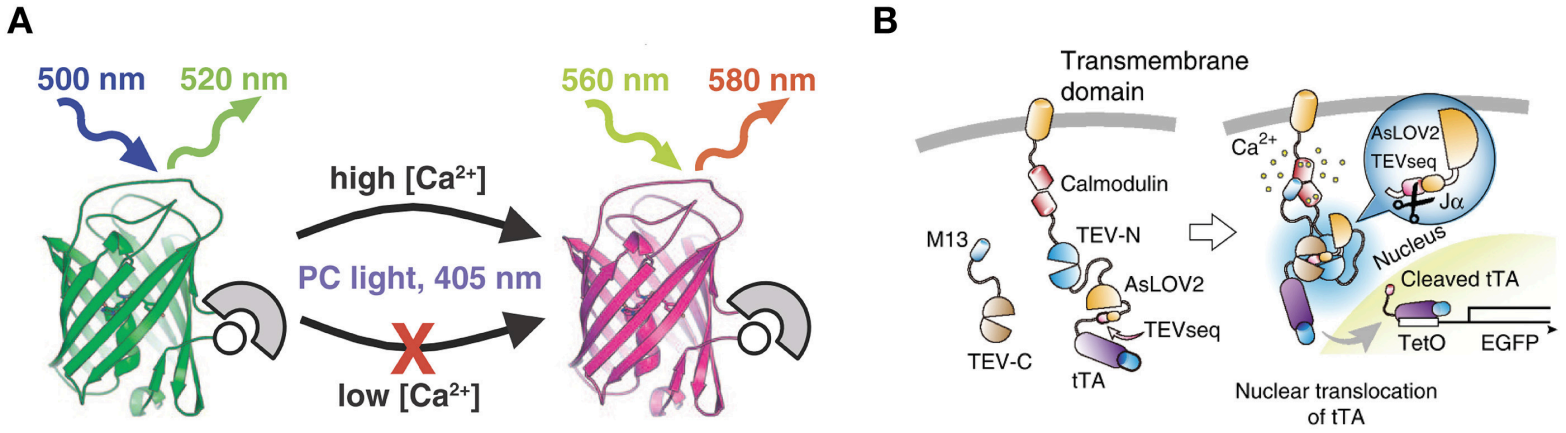
Schematic drawings of CaMPARI and Cal-Light. **(A)** CaMPARI, a calcium-modulated photoactivatable ratiometric integrator, is a circularly permuted fluorescent protein with calcium sensor domains that undergoes green-to-red photoconversion when exposed to ~400 nm light in the presence of high Ca^2+^. Adapted with permission from Fosque et al. (2015). **(B)** Cal-Light activation. When Ca^2+^ increases in the cytosol it triggers M13 and calmodulin to bind each other, allowing TEV-C and TEV-N to regain proteolytic functions. However, TEV protease cannot recognize TEVseq in a dark condition, because TEVseq is inserted at the C terminus of AsLOV2 Jα-helix. Blue light causes a conformational change in the Jα-helix, unmasking TEVseq. Cleaved tTA translocates to the nucleus and initiates gene expression. Adapted with permission from Lee et al. (2017).

CaMPARI is a bright green fluorescent protein that—via allosteric modulation of the chromophore—converts to a bright red fluorescent species upon illumination with violet light during high calcium availability (Fosque et al., 2015; Zolnik et al., 2017; Figure 1). The main advantage of CaMPARI over other genetically encoded calcium indicators (GECIs, Pologruto et al., 2004) is that photoconverted CaMPARI neurons are labeled irreversibly, allowing for imaging of an active network long after the photoconversion snapshot of activity has been obtained. Not only are active neurons marked but because CaMPARI is a ratiometric integrator, its red/green ratio indicates the level of their activity or, more precisely, the level of calcium influx. It is also a negative fluorescent indicator, meaning that it reports momentary calcium influx by a reduction in fluorescence intensity in both the unconverted green state and in the converted red state (see Supplementary Video Files 1, 2). A drawback with CaMPARI is that its expression is sensitive to tissue fixation using formaldehyde-based solutions. So while it is possible to track neurons in an *in vivo* experiment and follow them *in vitro*, it is not possible to make high quality images of the same neurons post-fixation (Zolnik et al., 2017). To overcome this problem, antibodies against a second generation of CaMPARI, CaMPARI2, have been designed to allow usage of immunocytochemistry to recover neurons that were marked *in vivo* (Moeyaert et al., 2018). In this study, except where explicitly stated otherwise, we used CaMPARI2.

### Mechanisms of Cal-Light Action

Cal-Light is light-sensitive and calcium-dependent (Lee et al., 2017). Activity-driven calcium increase in the cytosol is linked to gene transcription in the nucleus (Figure 1). To make this work, a tetracycline-controlled transcriptional activator (tTA) is tethered to the cell-membrane and the transcriptional activator is linked to a protease (Tobacco Etch Virus Protease, TEVp) cleavage sequence (TEVseq). Cleavage depends on blue light and calcium concentration. An increase in cytosolic calcium levels under presence of blue light leads to the release of tTA which then initiates gene transcription in the nucleus.

### General Rationale

**Figure 2:**
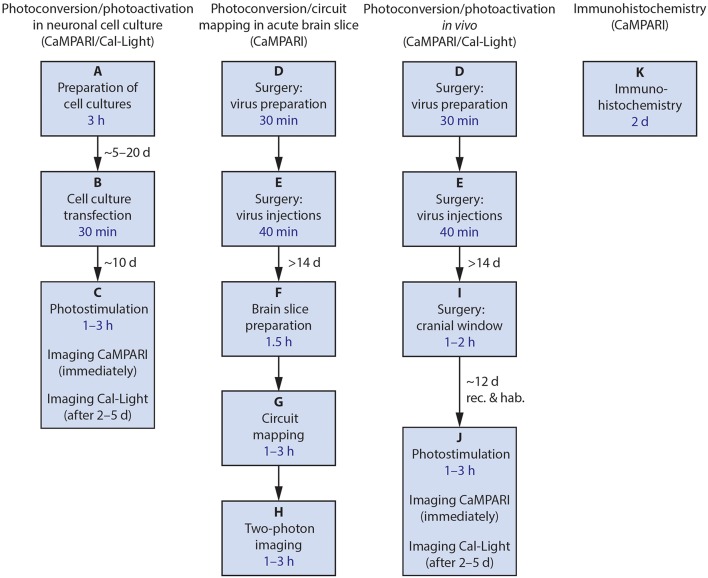
Flowchart of the four main procedures described. After each final process, confocal imaging and data analysis may be performed. Estimated amount of time needed for the main steps is included in the boxes in blue; necessary delays between major steps are given near the arrows. rec., recovery; hab., habituation.

The protocols presented here describe how we use these tools, and some of our modifications. The labs that developed these constructs have published papers demonstrating that they work *in vivo* in mice, and *in vitro* in tissue culture (Fosque et al., 2015; Lee et al., 2017) as well as in other model species including Drosophila and C. elegans (Fosque et al., 2015). Here, we describe the following procedures (Figure 2): (1) wide-field imaging for photoconversion and photoactivation in neuronal cell culture, (2) using CaMPARI and ChR2 *ex vivo* in acute brain slices for circuit mapping (Zolnik et al., 2017), (3) using both CaMPARI and Cal-Light *in vivo* in quietly sitting head-fixed mice as proof of concept experiments for monitoring large scale photoconversion and photoactivation with epifluorescence microscopy and *ex vivo* imaging, and (4) immunohistochemistry to recover CAMPARI2 expression in formaldehyde-fixed tissue.

### Rationale for Expressing CaMPARI and Cal-Light Constructs in Neuronal Cell Culture

**Figure 3:**
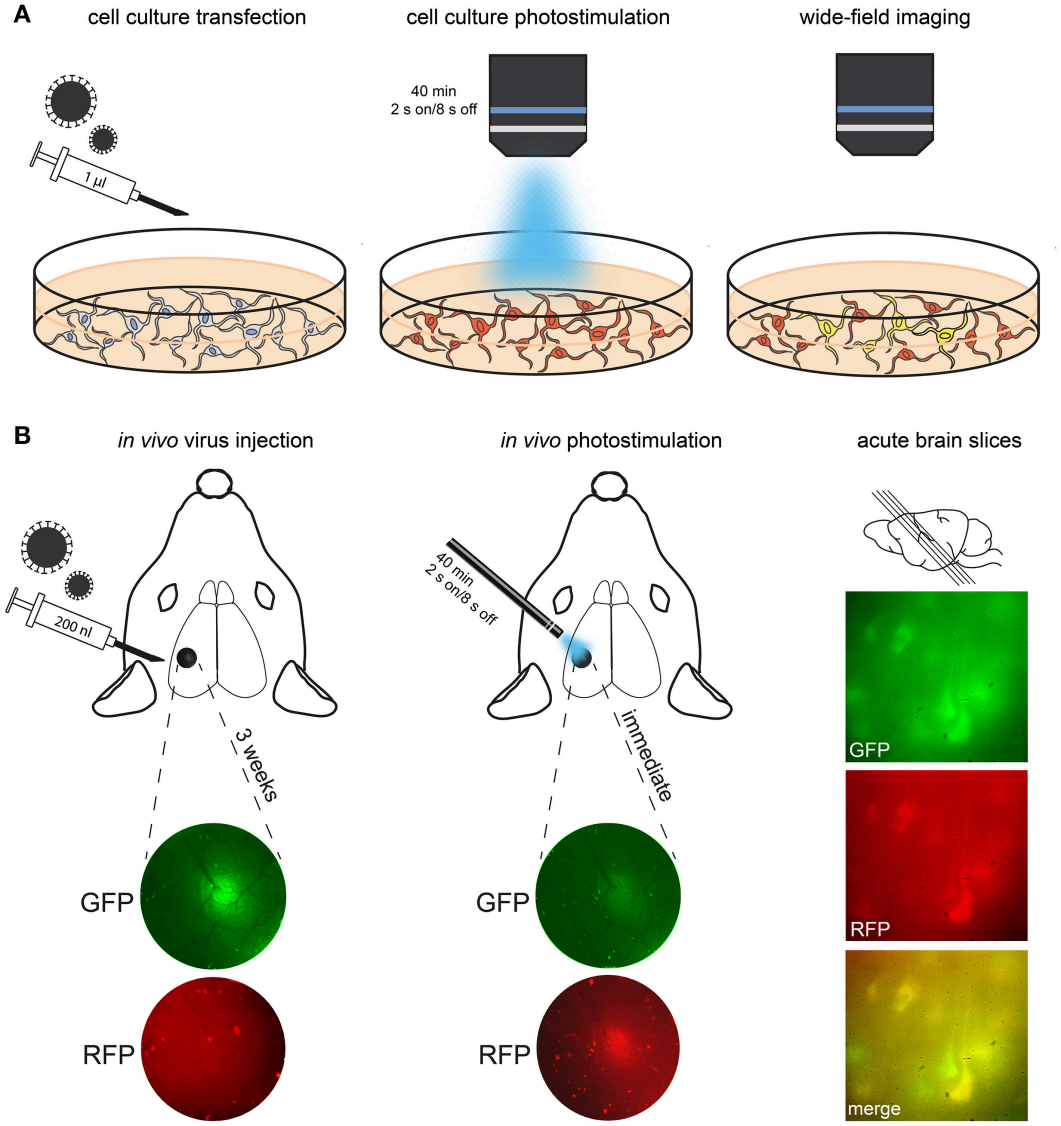
Procedure for transfection, conversion and imaging. **(A)** In this schematic, cultured neurons are treated with Cal-Light virus. After expression of the construct (indicated via red fluorescence), photostimulation is applied. Imaging then reveals neurons that were active while receiving the light stimulus (i.e., they co-express GFP and thus appear yellow in merged channels). **(B)** In this example, CaMPARI virus was injected into S1 cortex; expression of the construct is imaged using an epifluorescence microscope. A large number of neurons express CaMPARI 3 weeks after virus injection. Left, injection site imaged through the cranial window before and after 405 nm photoconversion. Note the reduction of green/red ratio immediately after photoconversion. Right, demonstration of photoconverted neurons in acute brain slices of S1 (parasagittal, 300 μm).

**Figure 4:**
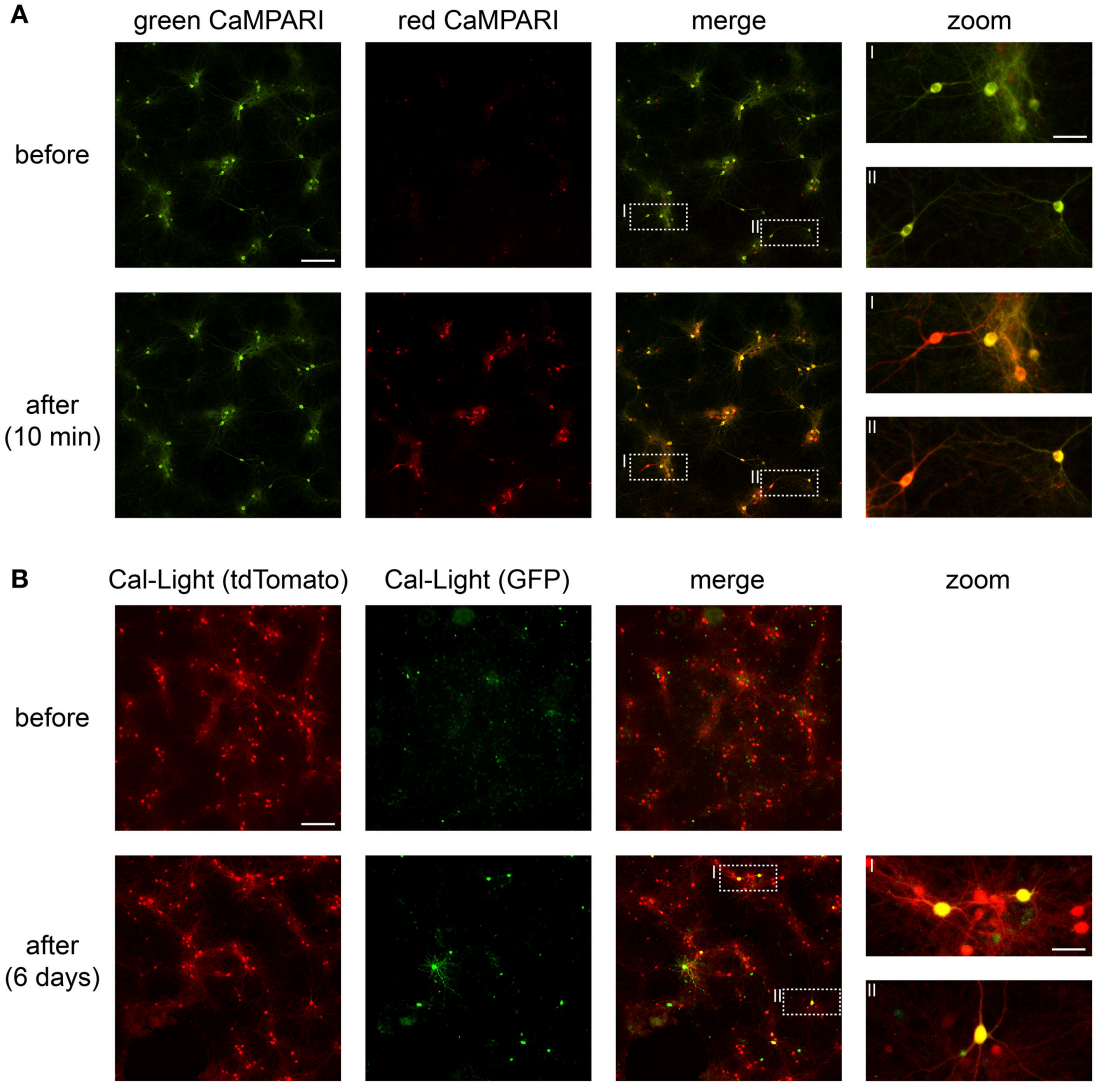
CaMPARI and Cal-Light expression and functionality in neuronal cell culture. **(A)** Wide-field epifluorescence images of neuronal cell culture expressing CaMPARI. Top row, pre-photoconversion images showing fluorescence exclusively in the green channel. Bottom row, post-photoconversion images showing mixed green and red fluorescence after ~5 min of total illumination with 395 nm light. **(B)** Wide-field epifluorescence images of neuronal cell culture expressing Cal-Light. Top row, pre-photoactivation images showing fluorescence in the red (tdTomato) channel. Bottom row, post-photoactivation images showing mixed red and green (GFP) fluorescence after ~8–10 min of total illumination with 470 nm light and after 6 days of expression time. Scale bars, 200 μm and 50 μm in insets. Images were taken on a wide-field epifluorescence microscope (Nikon Ti2) using a 10x air objective (Plan Apo, 0.8 NA, 1,000 μm WD) and a Lumencor Spectra X LED. Fluorescence was imaged through 519/26 nm (GFP) and 642/80 nm (tdTomato) bandpass emission filters.

The key requirement of photoconversion and photactivation is that calcium entry is coupled to exposure to light. However, the minimum duration of light exposure and the optimal timing of illumination and calcium entry are still not completely known. To measure the effectiveness of different light parameters, preparations such as dissociated neuronal culture combined with wide-field stimulation and live-cell imaging prove to be useful (Figures 3A, 4). Dissociated neurons can be routinely cultured from the cortex and hippocampus of postnatal day (P) 0–3 rat or mouse pups and can be maintained in culture for weeks to months. Although the network architecture is not conserved, dissociated neurons develop morphologically identifiable axons and dendrites, establish synaptic connections and fire repetitive trains of action potentials (Turko et al., 2019). Dissociated neurons are cultured in a monolayer, which makes them readily accessible to both optical and experimental manipulation. This accessibility is a distinct advantage over *ex vivo* and *in vivo* procedures, particularly when investigating the efficiency of viral transfection, gene expression and effectiveness of the light parameters. Dissociated cell cultures are therefore particularly well-suited for characterizing the expression and function of virus-mediated genetic manipulations.

### Rationale for Using Brain Slices

**Figure 5:**
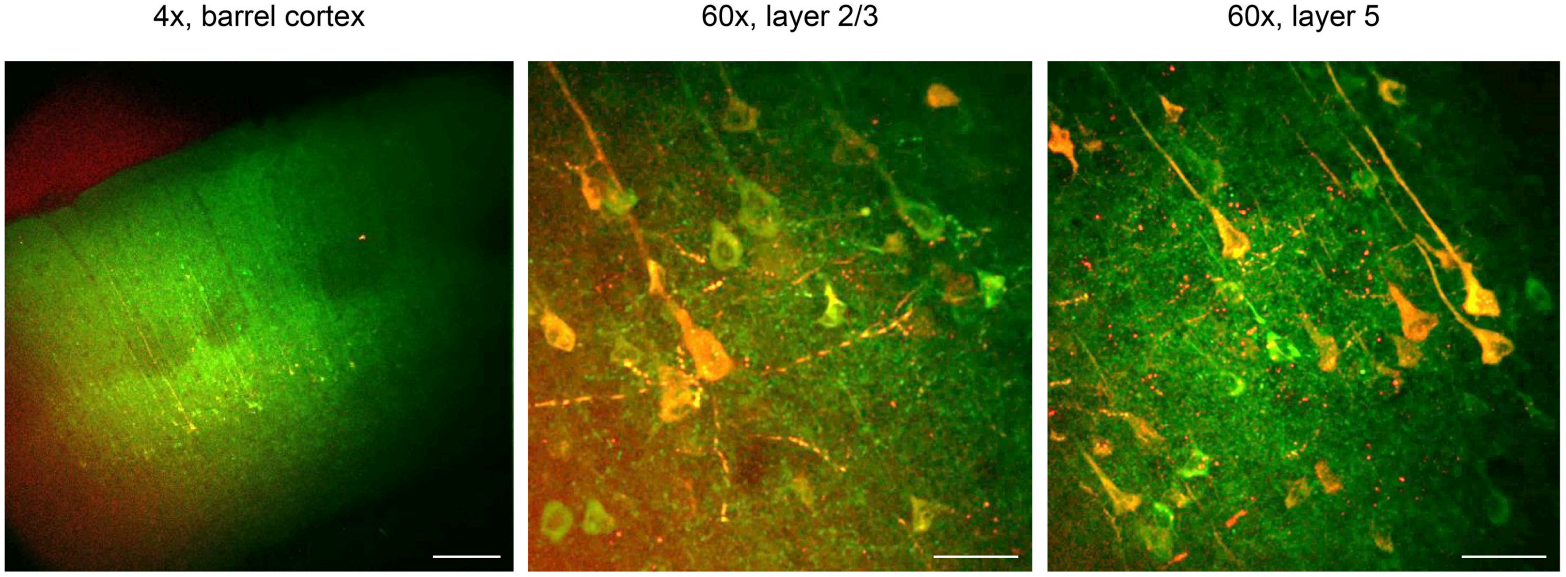
CaMPARI for all-optical functional connectivity mapping. CaMPARI was expressed in S1 and ChR2-EYFP was expressed in M1 cortex. In acute *ex vivo* brain slices, violet light (405 nm, 120 mW cm^−2^) was applied to S1 cortex that activates ChR2 and simultaneously drives conversion of post-synaptic CaMPARI-expressing neurons in layer 2/3 and 5. Here, conversion and stimulation light pulses were delivered at 10 Hz for a second, with a step of light for 5 s after the stimulus. There was a 12 s interval between each train, and this was repeated 10 times. Left, one-photon image of CaMPARI green/ChR2-EYFP under 4 × magnification. After violet light illumination, red CaMPARI fluorescence is evident in layers 2/3 and 5. Scale bar, 200 μm. Middle and right panels, post-stimulation/conversion 60 × magnification two-photon images of CaMPARI red/green in layers 2/3 and 5, from the corresponding slices. Scale bar, 50 μm. Exclusively in this Figure, CaMPARI (not CaMPARI2) was expressed.

A fundamental problem of all calcium sensors is to identify the cause and source of calcium entry. Cytosolic calcium levels rise when neurons fire action potentials, when synaptic activity depolarizes neurons, or when calcium is released from internal stores (Sabatini et al., 2002). Consequently, it is difficult to interpret calcium signals in experiments performed *in vivo*. In contrast, neurons are quiescent in brain slices and synaptic activity can be easily and specifically controlled. For the purpose of investigating the effectiveness of activity-tracking methods, we used *in vitro* experiments to establish that CaMPARI photoconversion was sensitive to changes in internal calcium caused by synaptic input, i.e., photoconversion did not depend on action potentials. Thus, in order to evaluate the CaMPARI signal, it was necessary to determine whether CaMPARI photoconversion occurred even when action potentials were blocked. For these kinds of experiments and for circuit mapping (Figure 5), brain slices are ideal due to the controlled conditions they offer: action potentials, synaptic transmission, and even intra- and extracellular conditions can be easily regulated using ionic composition and pharmacology.

### Rationale for *in vivo* Photostimulation and Epifluorescence Imaging Through a Cranial Window

**Figure 6:**
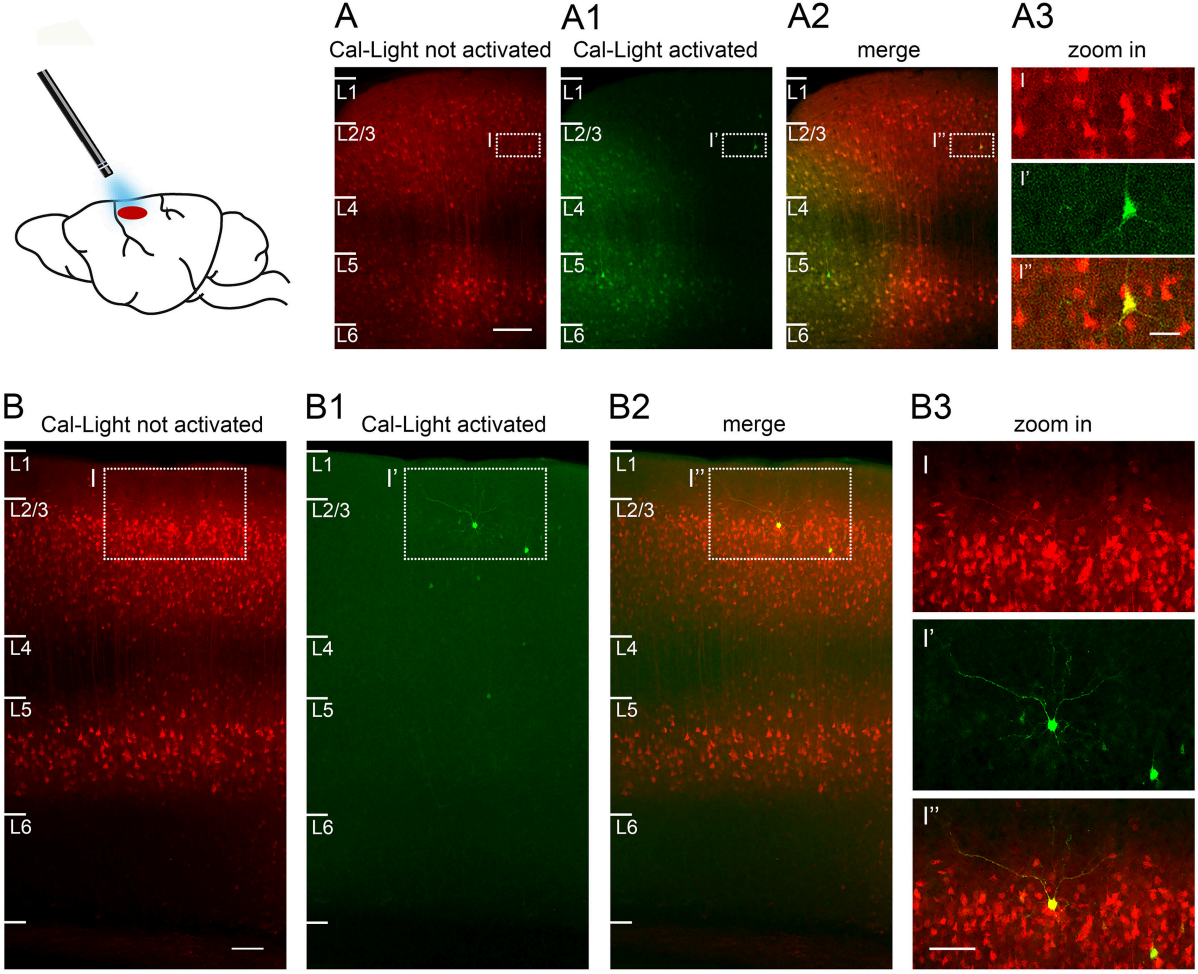
Sparse Cal-Light expression triggered through a cranial window in an awake quietly sitting head-fixed mouse. **(A)** A large number of neurons express Cal-Light three weeks after virus injection. **(A1)** Neurons from layer 1 to layer 4 express GFP six days after exposure to 470 nm light while the mouse was head-fixed and awake. A halo of GFP expression marks the extent of spread of the 470 nm light *in vivo*. **(A2)** Merged image showing photoactivated, double-labeled neurons (yellow) in layers 2–5. **(A3)** Magnified view of one strongly photoactivated neuron in layer 3. **(B)** Example of S1 cortex neurons expressing Cal-Light three weeks after virus injection. **(B1,B2)** Images are taken slightly off the center of injection to highlight the few intensely GFP expressing neurons in layers 1–4, 6 days after exposure to 470 nm light while the mouse was head-fixed and awake. **(B3)** Magnified view of one strongly photoactivated neuron (bright yellow) and one less strongly photoactivated neuron (pale yellow) in layer 2. Sections are parasagittal, 300 μm. Scale bar in A 100 μm, in B 200 μm, in insets 50 μm. Slices were imaged on a confocal laser scanning microscope (Nikon A1Rsi+) using a 20x air objective (Plan Apo, 0.8 NA, 1.000 WD) and a 647 nm laser. Fluorescence was imaged through a 700/50 bandpass emission filter.

The main purpose of these methods is to mark active neurons *in vivo* and to recover the neurons that were active for further analysis. Individual neurons can be tracked *in vivo* with two-photon imaging, while photoconverted regions of cortex can be tracked with epifluorescence. The principal advantage of epifluorescence is that measurements are simple, they take little time and the costs of wide-field epifluoresence microscopes are low compared to the costs of two-photon imaging setups (Andermann et al., 2013). The principal disadvantage is that the images are not at cellular resolution, nevertheless both Cal-Light-induced gene expression and CaMPARI photoconversion can be tracked using epifluorescence imaging through a cranial window (Figure 3B). Finally, neurons that were active *in vivo* can be imaged *ex vivo* (Figure 6) and characterized with respect to their physiology, anatomy, laminar distribution of their axon and dendrites as well as their expression of neurochemical markers. This offline approach means that the quantification of activity *in vivo* can be done more accurately without the need to keep the animal alive for extended periods.

### Rationale for Immunohistochemistry

**Figure 7:**
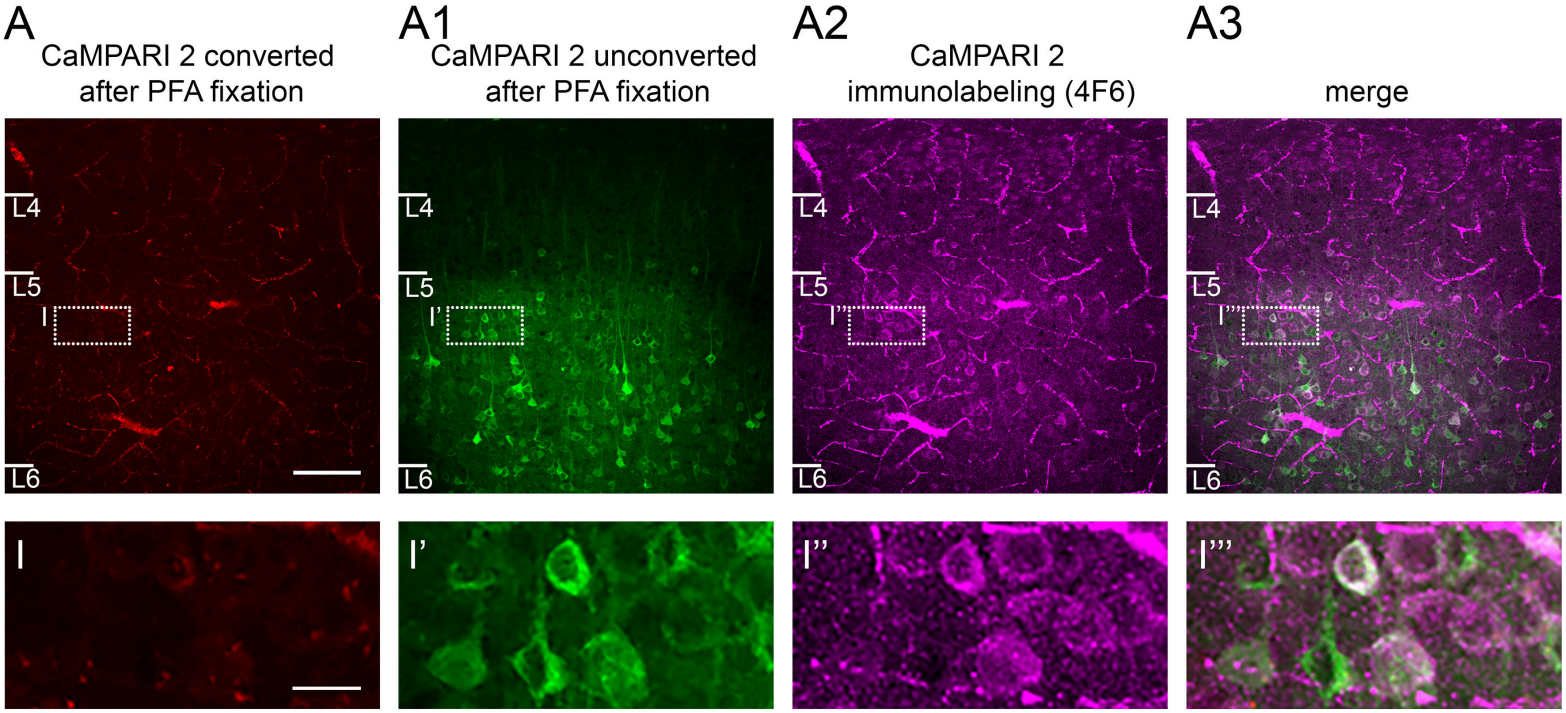
CaMPARI expression: Effects of fixation and use of immunostaining to recover converted neurons. **(A)** After fixation in 4% PFA, only a few neurons in S1 cortex show some weak red fluorescence signal reflecting the photoconversion of CaMPARI carried out *in vivo*. **(A1)** A large number of unconverted green neurons are still visible. **(A2)** When the CaMPARI 4F6 antibody targeting the converted form of CaMPARI is used, it reveals converted neurons, and **(A3)**, distinguishes them from neighboring unconverted neurons as seen in the merged image. Bottom row, magnified views of inset regions showing converted vs. non-converted cells. Sections are coronal, 300 μm and were taken directly from an *in vitro* experiment and placed in PFA overnight. Blood vessels are prominent because the brain was not perfused prior to fixation. Scale bar in A 100 μm, in inset 50 μm.

This method allows for recovery of CaMPARI-expressing neurons (including the photoconversion snapshot) in fixed tissue, opening up the possibility to mount sections and use them for confocal imaging at a later stage (Figure 7). In principle, this can then be used to identify and further characterize labeled neurons in terms of morphology and anatomical connectivity.

### Overview of the Procedures

We split procedures into several modules (Figure 2). For every experimental preparation, from tissue culture to *in vivo* work, the first step with both Cal-Light and CaMPARI is to obtain expression of the viral construct. Expression is then monitored through wide-field epifluorescence microscopy. Photoconversion or gene expression is triggered when light is applied to the cell culture, to the brain slice or through a cranial window onto the brain. In the case of *in vivo* procedures, neurons expressing the constructs can be recovered *ex vivo* for recording or circuit mapping. Finally, CaMPARI-expressing neurons that have been photoconverted and fixed in formaldehyde-based solution (and thus have lost their native fluorescence) can be recovered with immunohistochemistry.

## Materials and Equipment

### Animals

**Rats**. For neuronal cell cultures, we use wild type rat pups (Wistar, P0–2).

**Mice**. For circuit mapping, we use young (P21–30) wild type mice (C57/BL6J) of either sex. For *in vivo* photoactivation and photoconversion, we use adult (P40–90) mice of either sex.

### Chemicals

Neural Basal A media (ThermoFisher Scientific, 10888022)

B27 (ThermoFisher Scientific, 17504001)

Glutamax (ThermoFisher Scientific, 35050-038)

Penicillin-Streptomycin (10,000 U/ml, ThermoFisher Scientific, 15140-122)

Poly-L-Lysine (Sigma, P1399 Coverslip coating)

Papain (Sigma, P4762)

Bovine Serum Albumin (Sigma, A3294)

Hibernate A low fluorescence media (Brain Bits Ltd, HALF)

NaCl (Carl Roth, HN00.2)

KCl (Carl Roth, HN02.2)

NaH_2_PO_4_ (monohydrate, Carl Roth, K300.1)

NaHCO_3_ (Carl Roth, HN01.2)

CaCl_2_ (dihydrate, Carl Roth, HN04.2)

MgCl_2_ (hexahydrate, Carl Roth, HN03.1)

D-glucose (Carl Roth, HN06.3)

Choline chloride (Sigma, C7527)

Na-L-ascorbate (Sigma, A4034)

Na-pyruvate (Sigma, P2256)

KH_2_PO_4_ (Carl Roth, 3904.1)

NaOH (Carl Roth, 9356.1)

HCl (37%, Carl Roth, 9277.2)

Paraformaldehyde (Merck, 1.04005)

Gabazine (SR-95531 hydrobromide, Tocris, 1262)

Carbachol (carbamoylcholine chloride, Tocris, 2810)

Ethanol (96%, Carl Roth, T171.4)

Normal goat serum (Gibco, 16210-072)

Triton X-100 (Sigma, T8787)

Glycerol (Sigma, G5516)

DAPCO (Carl Roth, 0718.1).

### Drugs for Animal Use

Ketamine (10%, Medistart)

Xylazine (Xylavet, 20 mg/ml, CP-Pharma)

Isoflurane (Forene, Abbvie)

Buprenorphine (Temgesic, Reckitt Benckiser)

Carprofen (Rimadyl, Zoetis)

Lidocaine (Sigma, L7757).

### Solutions

**Neural Basal A (NBA) complete medium**. NBA medium, supplemented with B27 (at 1 × concentration), GlutaMAX (at 1 × concentration) and Penicillin-Streptomycin (100 U/ml).

**Hibernate a Complete Medium**. Hibernate A medium, supplemented with B27 (at 1 × concentration), GlutaMAX (at 1 × concentration) and Penicillin-Streptomycin (100 U/ml).

**ACSF**. (in mM): 125 NaCl, 2.5 KCl, 1.25 NaH_2_PO_4_, 25 NaHCO_3_, 2 CaCl_2_, 1 MgCl_2_, and 25 D-glucose in distilled water, pH ~7.4.

**Choline-ACSF**. (in mM): 110 choline chloride, 2.5 KCl, 1.25 NaH_2_PO_4_, 26 NaHCO_3_, 11.6 Na-L-ascorbate, 3.1 Na-pyruvate, 7 MgCl_2_, 0.5 CaCl_2_, and 10 D-glucose in distilled water, pH ~7.4.

**Sterile PBS**. (in mM): 137 NaCl, 2.7 KCl, 10 Na_2_HPO_4_, 1.8 KH_2_PO_4_ in distilled water. Adjust the pH to 7.4 using NaOH.

**Sterile Paraformaldehyde (4%)**. Dissolve 40 g of paraformaldehyde (PFA) in 800 ml of distilled water at 60°C. Add some drops of 1 M NaOH until the solution clears, and 100 ml of 10 × PBS. Adjust the pH to 7.4 with 1 M HCl. Adjust to 1,000 ml with distilled water and filter sterilize.

**Caution!** The formaldehyde is toxic and a known carcinogenic. Do not inhale, or come into contact with skin and eyes.

**Antibody and Blocking Solution**. Brain sections are treated with blocking solution (5% Normal goat serum (NGS), 1% Triton X-100 in 1 × PBS). Antibodies are incubated in the same solution.

**Mounting Solution**. 80% Glycerol + 2.5% DAPCO in PBS.

### Viruses/Antibodies

#### Viruses

All viruses were made in house by the Charité Viral Core Facility and were aliquoted and stored at −80°C. Aliquots of viruses in use (5 μl aliquots are typical) can be stored in a standard refrigerator at 4°C for several months. Viruses we used are:

- pAAV-TM-CaM-NES-TEV-N-AsLOV2-TEVseq-tTA
- pAAV-M13-TEV-C-P2A-TdTomato
- pAAV-TetO-GFP
- pAAV-Syn-CaMPARI2
- pAAV-ChR2-H134R-EYFP

#### Antibodies

- 1st antibody, CaMPARI 4F6, made at Janelia Farm Research Campus, Schreiter Lab, 1:1000
- 2nd antibody, Alexa 633, goat anti-mouse, Invitrogen A21050, 1:500

### Microscopes

Wide-field epifluorescence (used for cell cultures; Nikon Ti2)

Epifluorescence (used to check *in vivo* expression; Nikon Stereo SMZ1270i)

Confocal laser scanning (Nikon A1Rsi+)

Two-photon (Femto 2D two-photon laser scanning system, Femtonics Ltd, Budapest, Hungary).

### Surgical and Brain Slice Equipment

Dumont no. 5/45 cover slip forceps (Fine Science Tools, 11251-33)

Dumont no. 3, 4, 5, 7 forceps, assorted styles, straight (Fine Science Tools, 11231-30, 11254-20, 11241-30, 11251-10, 11271-30)

Standard-pattern forceps (Fine Science Tools, 11000-12, various lengths & diameters)

Spatula

Fine scissors (Fine Science Tools, 14060-09, 14058-09, 14090-09).

Dental drill (Osada Success 40 or Foredom Micromotor, HPA917).

Drill bits (Fine Science Tools, 19007-05, 19007-07, 19007-09)

Sterile single-use syringe, 0.4 ml (Omnican, B. Braun, 9161627)

Sugi absorbant swabs (Kettenbach Medical, 31602)

Parafilm (Sigma, P7793)

PCR Micropipettes, 1–5 μl (Drummond, 5-000-1001-X)

Eye care cream (Bepanthen, Bayer)

Mineral oil (Sigma, M3516)

Heating pad (Temperature Regulation System, FHC)

Self-adhesive resin cement (RelyX Unicem, 3M, Applicaps, 56815)

Contemporary Ortho-jet powder (black, Lang Dental, 1520BLK)

Kwik-Cast sealant (World Precision Instruments)

Pressure injector for low rate and small volume (Stoelting Quintessential Pressure injector, 53311)

Micropipette puller for virus-injection pipettes (Sutter Instrument, P-97)

Stereotaxic apparatus for small animals (KOPF, 940)

Vibratome (Leica, VT1200S)

### Illumination Equipment

Ti:sapphire laser (MaiTai HP DeepSee; Spectra-Physics/Newport)

455 nm LED (for *in vivo* stimulation; Prizmatix, UHP LED Head 455)

405 nm LED (for *in vivo* stimulation; ThorLabs, 405FP1e)

Optic fiber (for *in vivo* stimulation; Prizmatix, Optogenetics Fiber-500 *in-vitro*)

Mercury lamp (X-cite 200 W, Excelitas Technologies)

Optical power meter (ThorLabs, PM200 & S120VC)

### Software

For two-photon image acquisition: Matlab-based MES software package (Femtonics)

Image-processing software (ImageJ, https://imagej.nih.gov/ij/)

### Other

Eppendorf tubes, 0.5 ml (Sigma, T891)

Falcon tubes, 50 ml (Corning, 430921)

Glass coverslips (12 mm round; Roth, P231.1)

Glass coverslips (3–5 mm round; Warner Instruments, CS-3R-0, CS-4R, CS-5R-1)

Glass-bottom dishes (Eppendorf, 0030740017)

0.2 μm filters (Carl Roth, P668.1)

Haemocytometer (A. Hartenstein, ZK06)

24-well cell culture plates (Corning, 353047)

## Procedures

**(A) Preparation of dissociated primary neuronal cell cultures (duration: 3 h)**
  **Note:** The production of dissociated primary neuronal cell cultures has been described previously (Turko et al., 2019). All procedures should be performed under sterile conditions. All solutions should be filter sterilized using a 0.2 μm filter. Glass coverslips and dissection tools should be heat sterilized for 3 h at 185°C.
  1. Dissociate wild type cortico-hippocampal tissue from Wistar rat pups (post-natal days 0–2).
  2. Estimate cell densities using a haemocytometer or automated cell counter.
  3. Grow cells on 12 mm round-glass coverslips coated with Poly-L-Lysine (1 h coating; 20 μg/ml concentration).
  4. Plate cells in 24-well cell culture plates, at a density of 400 cells per μl in a 500 μl droplet (total cells per well: 2 × 10^5^).
  5. Culture cells in Neural Basal A medium, supplemented with B27 (at 1 × concentration), GlutaMAX (at 1 × concentration) and Penicillin-Streptomycin (100 U/ml). The incubator temperature should be 37°C.
  6. Feed cells weekly by removing 100 μl of conditioned cell culture medium and adding 200 μl of freshly made cell culture medium.
**(B) Cell culture transfection (duration: 20 min)**
  Prepare virus solutions in sterile aliquots. The total volume should be 1 μl per coverslip to be transfected.
  1. Wait 5–20 days for cultures to grow before infecting.
  2. Virus solutions:
    - For CaMPARI, prepare pAAV-Syn-CaMPARI2 with a titer of ~10^11^-10^12^ GC/ml.
    - For Cal-Light, mix three components:
      ° pAAV-TM-CaM-NES-TEV-N-AsLOV2-TEVseq-tTA
      ° pAAV-M13-TEV-C-P2A-TdTomato
      ° pAAV-TetO-GFP
    The pAAV-TetO-GFP can be replaced by viruses linking other genetic constructs, e.g., ChR2, iChloC, or ArCHT to TetO. GFP is expressed when pAAV-TetO-GFP is used. The ratio of the TetO construct to the two other viruses can be varied, depending on the experimental requirements (see Lee et al., 2017). Here, we use a mixture of 1:1:2 for the three viruses. The titers for each construct we used were ~10^12^-10^13^ GC/ml.
  3. Dilute the virus solution in 20 μl sterile PBS per 1 μl of virus. Vortex thoroughly.
  4. Take the well plate(s) out of the incubator and rapidly add 20 μl of the solution to each well in the plate containing coverslips to be transfected and then put them back into the incubator.
**(C) Photostimulation and imaging (duration: 1–3 h**, **Figure 4**, Supplementary Video Files 1, 2)
  To test CaMPARI photoconversion and Cal-Light photoactivation, cells have to be active and cytosolic calcium increases have to be coupled to light exposure. To promote network activity in cell cultures, cells were incubated with the GABA_A_ receptor antagonist gabazine blocking fast GABA-mediated synaptic inhibition. To further promote network activity, some cultures were also treated with the muscarinic receptor agonist carbachol.
  **Critical step:** After transfection, wait ~10 days for sufficient expression levels. If possible, check for expression of the constructs using an epifluorescence microscope. Optimally, cultures should be >21 days old at the time of photostimulation, leaving enough time for network maturation.
  1. Thaw frozen stock solutions of gabazine and carbachol at room temperature for 15 min. Incubate a conical tube containing 8 ml of hibernate A complete media in a water bath (37°C) for 15 min.
  2. Following incubation, pipette 2 ml of hibernate A complete media to a glass-bottom plate. Transfer a 24-well plate from the incubator to a laminar flow hood. Use sharp forceps to transfer a coverslip containing cells from the 24-well plate to the glass-bottom plate. Use a platinum ring (or suitable alternative) to hold the coverslip in place. Quickly transfer the 24-well cell culture plate back to the incubator.
    **Critical step:** We recommend using glass bottom dishes for imaging, as they offer improved optical quality.
    **Caution!** As hibernate A complete media is buffered for ambient CO_2_ levels, it is not compatible with incubators that are gassed with 5% CO_2_.
  3. Place the glass-bottom dish inside the microscope sample holder and focus on the cell layer. Depending on the experiment, drugs may be carefully pipetted into the hibernate solution to increase network activity. To reduce inhibition and to generate repetitive bursts of action potentials and increased network activity, apply 10 μM gabazine (final concentration) to cell cultures. If after 10 min gabazine does not increase network activity (activity can be monitored if cells express CaMPARI, see below), apply 10 μM carbachol (final concentration) to promote a further increase in activity. Take pre-stimulation images in any fluorescence channels that are of interest (see next step).
    **Note:** We use a Nikon Ti2 wide-field microscope designed for live-cell imaging.
  4. Image acquisition:
    - In cultures expressing CaMPARI, network activity can be monitored before photoconversion. This is possible because CaMPARI is also a calcium indicator related to GCaMP3, which dims rapidly and reversibly upon calcium influx (Fosque et al., 2015). Thus, changes in luminosity of CaMPARI-expressing neurons indicate that cells and circuits are active, with the dimming in fluorescence indicating an increase in intracellular Ca^2+^ concentration. A time series of 60 images at 1 Hz can be used to detect activity. Prior to photoconversion, there should be little to no fluorescent signal in the 555 nm channel.
    - In cultures expressing Cal-Light, images are acquired at 555 nm (to check tdTomato expression) and at 470 nm (to check GFP expression). In the pre-stimulation period, when 470 nm light has not been applied, there should be no fluorescent neurons in the green (470 nm) channel.
    **Note:** We used the following filters: 395/25 nm, 470/24 nm and 550/15 nm for excitation and 519/26 nm (GFP) and 642/80 nm (tdTomato) for emission.
    **Critical step:** If drugs are applied to the cultures, we recommend that light for photoconversion or photoactivation be delivered to the culture ~10 min after drug application. This ensures that there is sufficient time for diffusion of the drugs in the medium and develop their action on the neurons.
  5. Macros:
    - For CaMPARI, set up an automated macro script to acquire images in both 470 and 550 nm light channels, interspersed with 395 nm light stimulus (Supplementary Video Files 1, 2). We suggest acquiring a series of 60 images per channel, each followed by a 5 s pulse of 395 nm light, leading to 5 min of total light delivery. Carefully observe conversion and stop the procedure earlier if desired conversion levels are reached. The light power of the stimulus should be ~4–10 mW·cm^−2^.
    - For Cal-Light, set up an automated macro script that repetitively triggers a light stimulus at 470 nm (ON), followed by an interval of darkness (OFF). We suggest applying either 2 s ON/8 s OFF or 1 s ON/4 s OFF for 40–60 min, leading to 8–12 min of total light delivery. The light power of the stimulus should be ~4–10 mW·cm^−2^.
    **Critical step:** Due to its susceptibility to photobleaching, we recommend testing CaMPARI photoconversion at low light power first.
    **Note:** While CaMPARI photoconversion can be imaged immediately, photoactivation of Cal-Light takes ~2–5 days.
  6. Once photoconversion occurs or light for photoactivation of Cal-Light expression has been delivered, transfer the coverslip from the hibernate solution to a well-filled with cell culture medium in a laminar flow hood. Return the cell culture plate to the incubator.
  7. Further handling:
    - CaMPARI remains in its converted form for 2–3 days but will be progressively removed by protein turnover. In other words, the most reliable CaMPARI signal is detected right after conversion. Cells expressing CaMPARI can be used for additional experiments once protein turnover has removed the conversion, i.e., red CaMPARI has been fully replaced by newly produced green CaMPARI.
    - Cells expressing Cal-Light require at least 2–5 days to show reliable expression of the photoactivated construct. Make sure that incubator conditions (atmosphere and temperature) are optimal during that time and feed as required.
**(D) Surgical preparation for** ***in vivo* and** ***in vitro* expression of Cal-Light and CaMPARI: virus dilution, mixing, and loading (duration: 30 min)**
  1. Virus solutions:
    - Cal-Light: Prepare Cal-Light as described above [in section **(B)**(2)]. Mix three components:
      pAAV-TM-CaM-NES-TEV-N-AsLOV2-TEVseq-tTA,
      pAAV-M13-TEV-C-P2A-TdTomato and
      pAAV-TetO-GFP at a ratio of 1:1:2 (titers ~10^12^-10^13^ GC/ml)
    - CaMPARI: Prepare pAAV-Syn-CaMPARI2 with a final titer of ~10^11^-10^12^ GC/ml in a sterile 0.5 ml Eppendorf tube (total volume should be ~5 μl).
    **Critical step:** The optimal expression parameters of each virus should be determined in an initial step, where various virus dilutions series are tested.
    **Note:** Manual injections with pipettes or Hamilton syringes can be effective for injecting viruses, but here we use a motorized Quintessential injector (Stoelting). This injector is effective for controlled delivery of small volumes of virus. The flow rate and volumes (down to picoliters) can be adjusted. The injector is attached to a stereotaxic frame and pulled pipettes designed to deliver 5–10 μl, are positioned in the injector. 50–200 nl of virus are injected at 2–3 different depths at each injection site. Note that there are many methods for filling pipettes. It is possible to back-fill injection pipettes with mineral oil and then withdraw virus into the pipette tip, or to invert the process and fill the pipette tip with virus and back-fill with mineral oil.
  2. Pull glass pipettes for injections (5 or 10 μl) on a Sutter puller. Carefully cut the pulled pipettes back to ~10–20 μm with sharp scissors under a stereo-microscope. Before loading the virus, place a drop of mineral oil on a piece of sterile Parafilm.
  3. To load the virus, insert a pulled glass-micropipette tip-first into a plastic-pipetting tip attached to an insulin syringe. Make sure that there is no air leaking when negative pressure is applied on the syringe. Carefully back-load 300–500 nl of virus from an Eppendorf tube into the open end of the micropipette. After loading the virus, release the pressure on the insulin syringe. Position the plastic pipette opening over the prepared oil drop on the piece of Parafilm. Apply negative pressure on the syringe and load ~500 nl of mineral oil. The boundary between virus solution and mineral oil should be visible as a clear contrast of phases. Carefully remove the filled glass micropipette from the plastic syringe and place it into the injector attached to a stereotaxic arm. Dispose the Parafilm in a biohazard waste bin and keep the remaining virus solution in the refrigerator at 4°C.
    **Critical step:** Ensure that the tip of the micropipette is not damaged and that it has the correct size (diameter ~10 μm). This precaution should be taken to avoid damaging of dura and brain tissue.
**(E) Surgical preparation for** ***in vivo* and** ***in vitro* expression of Cal-Light and CaMPARI: stereotaxic injections (duration: 40 min)**
  **Critical step:** Clean the surgery area and the surgical instruments with 70% ethanol and let dry. If possible, to prevent any infections, surgical instruments can be autoclaved and sterile packs can be prepared for use in surgery.
  1. Before surgery, anesthetize mice deeply with an intraperitoneal injection of ketamine/xylazine (100/10 mg kg^−1^) solution. Once mice no longer react to tail or toe pinches, trim the fur on the head with sharp scissors or with an electric trimmer. Place mice into the stereotaxic apparatus (Kopf Instruments Inc., California, USA). Make sure that ear bars and the mouth piece are positioned correctly to hold mice in place for the duration of the surgery.
    **Caution!** All experiments with animals must have an animal license number and be approved by institutional and/or governmental agencies. The experiments described in this protocol were conducted after approval by the Landesamt for Gesundheit und Soziales (LAGeSo), Berlin, Germany.
    **Critical step:** It is absolutely crucial that the mice are well-anesthetized and receive the correct doses of analgesics.
  2. Provide local analgesia by injecting lidocaine (1–2%, 0.1–0.2 ml) locally under the scalp where the craniotomy is to be made.
    **Caution!** Apply eye care cream (Bepanthen) to prevent the eyes from drying out.
  3. When mice are fully sedated and positioned properly in the stereotaxic frame, ensure that the head is leveled and aligned. For injections deep into the brain it is necessary to ascertain that the *Z*-plane, the anterior and posterior parts of the skull are flat, positioned completely horizontally. Flat plane skull can be ascertained by positioning the micropipette tip at bregma, measuring the *z*-position, and repeating this measurement at lambda. Head fixation can be adjusted until the readings at the two points on the skull are identical.
  4. Once the fur is trimmed, disinfect the scalp using 70% ethanol. Carefully cut the scalp with a sterile scalpel and the splay the skin out with a forceps (Dumont, no 5). If needed, irrigate the wound edge with saline. Remove any excess liquid using absorbent swabs (Sugi, Kettenbach).
  5. Define the stereotaxic coordinates by setting the reference point “0” at bregma. Mark the cortical area of interest (in our case, barrel cortex, medial-lateral 2.5 mm, anterior-posterior −2.0 mm) with a pen or carefully with a scalpel blade. Drill a circular craniotomy of 1 mm radius around the mark. Apply careful and slow drilling without fully perforating the bone. By constantly applying sterile PBS to the bone, heating, and damaging of the dura can be avoided. After thinning the bone by continuous drilling, it should be possible to remove the perforated piece of bone.
    **Critical step:** While drilling, carefully check the thickness of the remaining bone. Avoid damaging the dura or the brain by applying too much pressure or by overheating the drill bit by persistent drilling. Regular irrigating with saline solution or air cooling is essential!
  6. Apply sterile PBS onto the craniotomy and carefully clean the injection site using absorbent swabs. Keep the brain moist with PBS.
  7. Put the virus-filled injection micropipette into the stereotaxic holder and place the pipette over the injection site in a 90° angle to the brain. Use the micro-injection controller to apply positive pressure and to generate a small drop of virus, visible at the tip of the pipette. This is to assure that the pipette is not clogged and virus solution can be injected smoothly.
  8. Lower the injection micropipette and penetrate the dura to reach the desired depth (in our experiments, we injected at 0.6, 0.4, 0.2, and 0.1 mm) below the pial surface. Once the pipette is at its correct position, the virus can be injected with positive pressure (100–200 nl at 15–20 nl min^−1^).
    **Critical step:** Ensure that the micropipette is not clogged and that the dura is moist so that the brain is not damaged when penetrating the pia with the injection pipette.
  9. After injection, wait for 5 min before removing the micropipette slowly from the cortex. Remove the pipette from the stereotaxic holder and dispose in a biohazard waste bin. Carefully clean the brain again with sterile PBS.
    **Note:** If CaMPARI circuit mapping [procedure **(G)**] is planned, perform another virus injection using ChR2 in the presynaptic area of interest.
  10. Inject the analgesics carprofen (5 mg/kg) and buprenorphine (0.05–0.1 mg/kg) intraperitoneally to ensure a pain-free recovery of the animal.
  11. Remove the mouse from the stereotaxic frame by loosening the ear bars and the nose piece.
  12. Suture the scalp with sterile suture sewing thread. Carefully put the mouse back into its home cage, which is put on a warming device. Monitor the mouse until it has woken up.
    **Critical step:** Ensure that the mouse is waking up under smooth conditions. Avoid placing an anesthetized mouse together with awake mates in one cage.
    **Caution!** After surgery, care has to be applied to each mouse individually and according to the regulations and guidelines. The mice of our experiments were monitored daily for 3 days after the surgery for pain, divergent behavior in food uptake, and abnormal social behavior.
**(F) Acute brain slice preparation (duration: 1.5 h)**
  1. Allow CaMPARI to express for >14 days following viral injection(s) *in vivo*.
  2. Deeply anesthetize the mouse (postnatal age >P21) with isoflurane (1.5–3% in O_2_). Remove and section the brain into coronal, 300 μm thick slices with a vibratome under cold (~0°C) choline-based artificial cerebrospinal fluid (ACSF).
    **Note:** For brain slicing and initial incubation (≤5 min), the use of choline-ACSF may help to improve slice viability. For photoconversion and recording, use normal ACSF.
  3. Transfer each slice after sectioning to an incubation chamber at 32°C for 5 min in a solution containing choline ACSF saturated with 95% O_2_/5% CO_2_.
  4. Transfer the slices into an incubation chamber containing normal ACSF at 32°C for 25 min and then at room temperature for an additional 30 min before use in experiments.
    **Note:** Brain sections stored in ACSF saturated with 95% O_2_/5% CO_2_ at room temperature remain viable for up to ~6–8 h.
**(G) Circuit mapping** ***in vitro* with CaMPARI (duration: 1–3 h**, **Figure 5****)**
  CaMPARI can be used to map cortical circuit activity driven by optogenetically defined inputs in brain slices (Zolnik et al., 2017). After slicing, axon terminals remain functional and excitable by light when expressing ChR2 (Petreanu et al., 2007; Cruikshank et al., 2010), enabling an *in vivo*-like assessment of specific input pathways. Additionally, neurons in acute brain slices are normally hyperpolarized and minimally active, which provides a low background for more reliable signal. As a planar section, the brain slice can be uniformly illuminated, eliminating confounds from uneven illumination intensity. The resulting post-synaptic activation pattern in an acute brain slice reflects functional connectivity from the target pathway. Control experiments with the same stimulation and light conditions can be performed to measure background conversion from green to red in the absence of ChR2 expression (see Figure 4 in Zolnik et al., 2017).
  **Note:** A range of filters and stimulation parameters can work for circuit mapping with ChR2 and CaMPARI. For example, a Cy3 emission filter (~580/50 nm) includes the peak fluorescent emission of CaMPARI red (Fosque et al., 2015). However, even a 650/50 nm filter works for imaging CaMPARI red, despite its very red shifted emission band (Zolnik et al., 2017). For imaging CaMPARI green, a FITC filter (~475/35 nm), appropriate for GFP or GCaMP imaging is ideal.
  1. Deliver violet light for optogenetic stimulation and CaMPARI photoconversion using an X-cite 200 W mercury lamp (Excelitas Technologies, Mississauga, Ontario, Canada) and light guided through a 405/10 nm bandpass filter (Semrock, FF01-405/10-25). In one-photon imaging experiments, photoconversion/stimulation light is delivered by a UPlanFL 4 × /NA 0.13 objective.
    Measure the light stimulus intensity with a Thor Labs optical power meter (PM 200) and a photodiode sensor that works in the UV range (S120VC). For the experiments in Figure 5, the light power was 120 mW cm^−2^ at 405 nm. This light intensity is sufficient for inducing conversion and activating ChR2. The stimulation/conversion and initial fluorescence imaging was performed under a one-photon microscope through a 4 × objective.
    **Critical step:** Before applying the stimulation protocol, it is necessary to acquire baseline images in both red and green wavelengths.
    **Critical step:** In case slices are to be used for immunocytochemistry [procedure **(K)**], live imaged brain sections should be marked prior to fixation—this is essential for aligning the *post-hoc* image to the anti-CaMPARI immunostained section.
    **Note**: ChR2 is not efficiently activated at 405 nm (peak activation ~470 nm), and therefore higher light intensities may be necessary or additional light must be delivered at 470 nm to boost the ChR2 excitation. If using stronger 405 nm light to activate ChR2, this will increase the CaMPARI conversion rate (Zolnik et al., 2017) and thus the duration of illumination may need to be adjusted.
  2. Photoconvert neurons with 10 pulses, 15 ms in duration, delivered at 10 Hz, followed by a 5 s-long light pulse. This protocol—especially the 5 s light pulse at the end of the stimulus train—ensures that the photoconversion light is delivered when calcium is elevated in the post-synaptic target neurons.
  3. For quantification of the red/green ratios of each neuron, two-photon imaging is necessary. A standard brain slice immersion chamber is needed to maintain slice viability during live imaging for these experiments.
    **Note:** Optimally, the photoconversion and imaging steps can be combined by delivering 405 nm light through the objective used on a two-photon imaging setup. However, it is also possible to deliver light obliquely to the sample by using the light guide, filter, and a collimator (Cairn Research, Faversham, UK) and lens (Thorlabs, Newton, NJ, USA; AC254-030-A-ML, *F* = 30 mm).
**(H) Two-photon imaging (duration: 1–3 h**, **Figure 5****)**
  1. Use a two-photon laser scanning system equipped with a femtosecond pulsed Chameleon Ti:Sapphire laser controlled by the MES software package.
  2. Tune the laser to λ = 820 nm for excitation of CaMPARI red and green fluorescence.
    **Note:** If you have access to a two-photon system with a widely tunable laser, we would recommend imaging CaMPARI green at λ = 980 nm and CaMPARI red at λ = 1,040 nm.
  3. Detect fluorescence in epifluorescence mode with a water immersion objective (LUMPLFL 60 × /1.0 NA, Olympus, Hamburg, Germany), and trans-fluorescence and transmitted infrared light with an oil immersion condenser (Olympus; 1.4 NA). Emission light can be divided with a dichroic mirror at ~590–600 nm, and green and red signals filtered using 525/50 and 650/50 bandpass filters, respectively.
    **Note:** The emission of CaMPARI red peaks at ~580 nm, and thus a filter that contains this fluorescence band is ideal, but be careful to check that there is no bleed through from the excitation of the CaMPARI green.
    **Critical step:** Carefully handle the slices when placing them in the recording chamber to maintain cell viability during photoconversion.
    **Caution!** Ensure that the slice remains in a stable position before starting z-stack image collection. Slice movement can distort your images.
**(I) Surgical preparation for** ***in vivo* expression of Cal-Light and CaMPARI: cranial window and head post-implant (duration: 1–2 h)**
  1. Allow >14 days for expression of constructs after surgery.
  2. Follow steps (1)–(4) of module **(E)**.
  3. Incise the scalp and scrape the skull. Carefully remove the fascia and let the skull air dry. When dry, place a metal head post (ours are custom-made, the shape varies) on the clean and dry skull. Next, use self-adhesive resin cement (RelyX Unicem, 3M ESPE) to glue the head post in place.
  4. Once the cement has hardened, drill a circular craniotomy of 3 mm radius around the spot where virus had been injected previously. Apply careful and slow drilling without fully perforating the bone. By constantly applying sterile PBS to the bone, heating and damaging of the dura can be avoided. After thinning the bone by continuous drilling, it should be possible to remove the perforated piece of bone.
    **Critical step:** While drilling, carefully monitor the thickness of the underlying bone. Avoid damaging the dura or the brain by applying too much pressure or by drilling continuously and overheating the drill bit and brain. Irrigate using sterile saline/PBS if needed and remove excess liquid with absorbent swabs.
  5. Prepare a 3 mm glass coverslip by cleaning in 70% ethanol. Carefully place the coverslip on the part of the mouse brain that is uncovered by the craniotomy. Use the arm on the stereotaxic apparatus to position a wooden tipped applicator or toothpick over the coverslip and apply slight pressure to push the coverslip into the craniotomy and hold in place.
  6. Apply superglue at the edges of the coverslip and bone to fix the coverslip into the craniotomy, then wait for the glue to dry and carefully release the wooden tip off the window.
    **Critical step:** Be careful not to drop glue on top of the glass window as this will reduce the imaging quality.
  7. Cover the head post using Ortho-jet powder (Lang Dental, Black) to fill any gaps and to increase stability.
  8. Cover the glass and well-around the craniotomy with Kwik-Cast Sealant silicone (WPI, World Precision Instruments).
  9. Once the sealant firmed up, apply analgesics [see procedure **(F)**], take the mouse out of the stereotaxic apparatus and monitor. Once the mouse wakes up, place it back in its home cage.
**(J)** ***In vivo* photostimulation (duration: 1–3 h;** **Figures 3B**, **6****)**
  **Critical step:** Allow >7 days for mice to recover from head post and cranial window surgery. Habituate mice by handling and head-fixing them for increasing durations in several sessions. Reward (e.g., sweet milk) may be given to mice to make them more comfortable during the habituation process. In head-fixed mice, check for expression of the construct and take an image using an epifluorescence microscope.
  **Critical step:** Re-apply Kwik-Cast silicone whenever the sealant is lost or removed for microscopy to avoid possible unwanted photostimulation via ambient light sources.
  1. For the main photostimulation session, head-fix the mouse in the stimulation setup and remove the sealant. Place the tip of an optic fiber connected to the LED light source directly on top of the glass window at a 90° angle. Make sure that the light spot overlaps with the area of expression.
    **Caution!** If the mouse appears to be in pain or severe discomfort during the session, stop the experiment and extend the habituation period.
  2. Photostimulation.
    - For Cal-Light-expressing animals, set up an automated macro script that repetitively triggers a light stimulus at 470 nm (ON), followed by an interval of darkness (OFF). We suggest applying either 2 s ON/8 s OFF or 1 s ON/4 s OFF for 40–60 min, leading to 8–12 min of total light delivery. The light power of the stimulus should be ~20–30 mW (measured at the tip of the optic fiber).
    - For CaMPARI, set up an automated macro that repetitively triggers a light stimulus at ~395–405 nm (ON), followed by an interval of darkness (OFF). We suggest applying either 2 s ON/8 s OFF or 1 s ON/4 s OFF for 40–60 min, leading to 8–12 min of total light delivery. The light power of the stimulus should be ~20–30 mW (measured at the tip of the optic fiber).
    **Caution!** Measure light intensity at the fiber tip and use light intensity at surface of <30 mW and carefully monitor light application, as high light power may cause damage on the brain!
    **Note:** Cal-Light-triggered gene expression takes 2–5 days. CaMPARI photoconversion occurs within seconds.
**(K) CaMPARI immunohistochemistry (duration: 2 days;** **Figure 7****)**
  1. After imaging of the live brain slices, fix them in formaldehyde-based (4%) fixative at 4°C overnight.
    **Critical step:** After formaldehyde fixation, an image can be taken for control that no endogenous expression is left.
    **Critical step:** Be careful not to confound sides of brain slice (upper, converted vs. lower, non-converted side). This is important for later analysis at the confocal microscope.
  2. Wash slices in phosphate buffered saline solution (PBS) and block in blocking solution for 2 h at room temperature.
    **Critical step:** Rinse slices in PBS thoroughly to ensure that compounds are washed out.
  3. Incubate brain slices in primary antibody containing solution (CaMPARI 4F6, at a dilution of 1:1,000 in blocking solution) at 4°C overnight.
  4. Dispose the primary antibody, rinse slices in PBS before incubation in the secondary antibody solution (Alexa 633, goat anti mouse, Invitrogen A21050, at a dilution of 1:500 in blocking solution) for 2 h at room temperature.
  5. Dispose the secondary antibody, rinse slices in PBS.
  6. Mount slices in mounting solution (80% glycerol + 2.5% DAPCO in PBS) on a regular slide and coverslip.
    **Note:** An immunostaining for endogenous CaMPARI in blue (405 nm excitation wavelength) can be added with the Flag antibody (Sigma F425), which works nicely in cultured neurons.

## Anticipated Results

### Photostimulation in Neuronal Cell Culture

After transfection, cultured neurons begin to express CaMPARI within ~10 days, this appears as a green fluorescent signal. Calcium transients in these neurons are noticeable as dimming in fluorescence and occur when network activity increases (e.g., via blocking inhibition and/or by application of carbachol; see Supplementary Video Files 1, 2). Active neurons expressing CaMPARI photoconvert from green to red when 395 nm light is applied during active states (Supplementary Video Files 1, 2 and Figure 4).

Cultured neurons express Cal-Light within ~10 days, indicated by a red fluorescent signal via tdTomato expression. When network activity increases (e.g., by blocking inhibition and/or by application of carbachol), active neurons expressing Cal-Light are photoactivated and express GFP within 2–5 days after exposure to 470 nm light (Figure 4).

### Circuit Mapping in Acute Brain Slices

Two to three weeks after virus injection, neurons begin to express CaMPARI. At this point, *ex vivo* brain slices can be prepared in which CaMPARI green-expressing neurons should be visible in both single-photon and two-photon excitation. Circuit mapping experiments, by applying 405 nm light, then reveal post-synaptic targets of axon fibers expressing ChR2 via photoconversion of CaMPARI from green to red (Figure 5).

### Photostimulation *in vivo*

Two to three weeks after virus injection, neurons begin to express Cal-light or CaMPARI. This expression (green CaMPARI or red Cal-Light) is visible through the cranial window using epifluorescence microscopy (Figure 3B). At this point, photostimulation is applied *in vivo* in head-fixed, habituated quietly sitting mice. After photostimulation, a shift from green to red fluorescence should appear within seconds in the case of CaMPARI (Figure 3B) or additional green fluorescence should appear after 2–5 days in the case of Cal-Light. Preparation and imaging of brain slices or sections will then reveal individual neurons that were labeled (Figures 3B, 6).

### Immunohistochemistry

Immunostaining recovers the CaMPARI red signal in photoconverted neurons that is quenched after fixation (Figure 7). Following the immunostaining steps, single converted and non-converted neurons are distinguishable based on their expression, conversion, laminar localization and other anatomical features.

## Data Availability

All datasets generated for this study are included in the manuscript and/or the Supplementary Files.

## Ethics Statement

We performed all procedures in accordance with protocols approved by the Charité—Universitätsmedizin Berlin and the Berlin Landesamt für Gesundheit und Soziales (LAGeSo) for the care and use of laboratory animals.

## Author Contributions

CE, SD, PT, PP, BE, IV, ML, and RS designed the study. CE, JL, TZ, SD, and AP did the experiments. CE, JL, PT, and RS wrote the paper. All authors read and edited text, contributed to manuscript revision and approved the submitted version.

### Conflict of Interest Statement

The authors declare that the research was conducted in the absence of any commercial or financial relationships that could be construed as a potential conflict of interest.

## Supplementary Material

Supplementary Video Files 1 and 2 Photoconversion of CaMPARI-expressing cells in neuronal cell culture using wide-field epifluorescence imaging. Momentary dimming of fluorescence indicates calcium-intensive events. Videos contain 60 frames, each taken prior to a 5-s long pulse of 395 nm light. Green and red imaging channels are merged. Video 1, using 10 × objective and Video 2, using 20 × objective.
